# Prediction of stent under-expansion in calcified coronary arteries using machine learning on intravascular optical coherence tomography images

**DOI:** 10.1038/s41598-023-44610-9

**Published:** 2023-10-23

**Authors:** Yazan Gharaibeh, Juhwan Lee, Vladislav N. Zimin, Chaitanya Kolluru, Luis A. P. Dallan, Gabriel T. R. Pereira, Armando Vergara-Martel, Justin N. Kim, Ammar Hoori, Pengfei Dong, Peshala T. Gamage, Linxia Gu, Hiram G. Bezerra, Sadeer Al-Kindi, David L. Wilson

**Affiliations:** 1 Department of Biomedical Engineering, Faculty of Engineering, The Hashemite University, Zarqa, Jordan; 2 Department of Biomedical Engineering, Case Western Reserve University, Cleveland, OH 44106 USA; 3 Cardiovascular Imaging Core Laboratory, Harrington Heart and Vascular Institute, University Hospitals Cleveland Medical Center, Cleveland, OH 44106 USA; 4 Department of Biomedical and Chemical Engineering and Sciences, Florida Institute of Technology, Melbourne, FL 32901 USA; 5 Interventional Cardiology Center, Heart and Vascular Institute, University of South Florida, Tampa, FL 33606 USA; 6 Department of Radiology, Case Western Reserve University, Cleveland, OH 44106 USA

**Keywords:** Interventional cardiology, Cardiology, Biomedical engineering

## Abstract

It can be difficult/impossible to fully expand a coronary artery stent in a heavily calcified coronary artery lesion. Under-expanded stents are linked to later complications. Here we used machine/deep learning to analyze calcifications in pre-stent intravascular optical coherence tomography (IVOCT) images and predicted the success of vessel expansion. Pre- and post-stent IVOCT image data were obtained from 110 coronary lesions. Lumen and calcifications in pre-stent images were segmented using deep learning, and lesion features were extracted. We analyzed stent expansion along the lesion, enabling frame, segmental, and whole-lesion analyses. We trained regression models to predict the post-stent lumen area and then computed the stent expansion index (SEI). Best performance (root-mean-square-error = 0.04 ± 0.02 mm^2^, *r* = 0.94 ± 0.04, *p* < 0.0001) was achieved when we used features from both lumen and calcification to train a Gaussian regression model for segmental analysis of 31 frames in length. Stents with minimum SEI > 80% were classified as “well-expanded;” others were “under-expanded.” Under-expansion classification results (e.g., AUC = 0.85 ± 0.02) were significantly improved over a previous, simple calculation, as well as other machine learning solutions. Promising results suggest that such methods can identify lesions at risk of under-expansion that would be candidates for intervention lesion preparation (e.g., atherectomy).

## Introduction

Patients with inadequate stent expansion are at high risk for adverse outcomes, including stent thrombosis and in-stent restenosis^1^. Both are well-described complications that often cause acute coronary syndromes and, in the worst-case scenario, sudden cardiac death. Despite substantial improvements made in interventional procedures, stent design, drugs, and polymers as well as the adoption of therapeutic strategies, acute stent thrombosis, and in-stent restenosis remain critical issues^2^. It can be difficult/impossible to fully expand a coronary artery stent in a heavily calcified coronary artery lesion. Once a stent is implanted in atherosclerotic tissue that is highly resistant to dilation, there is no opportunity to apply a pre-stent treatment option such as atherectomy. The only option is to use post-stent, high-pressure (up to 30 atm) balloon dilations. Careful evaluation of these risks of under-expansions before the intervention will aid treatment planning, including the potential application of a pre-stent plaque modification strategy. Software for predicting stent expansion could play a fundamental role in the clinical management of patients, with important implications regarding the choice of optimal medical therapy.

In percutaneous coronary intervention (PCI) planning, intravascular optical coherence tomography (IVOCT) is a useful tool for identifying lesion severity, reference vessel size, lesion length, and the extent of calcification^2, 3^. IVOCT-guided stent implantation has been shown to significantly improve clinical outcomes as compared with angiographic-only guidance. IVOCT imaging provides a detailed pre-stent evaluation of lesion morphology, including calcification and lipidous regions, and provides a detailed analysis of stent deployment characteristics (e.g., stent expansion, malapposition, and stent edge dissection)^4^. Quantitative assessments of stent expansion such as stent expansion index (SEI) are possible with IVOCT.

In response to the need to predict under-expansion, in a landmark paper, Fujino et al. proposed an IVOCT-based calcium score to predict stent expansion and the need for pre-stent plaque modification^4^. Matsuhiro et al. investigated whether several calcium parameters were correlated with stent expansion in moderate calcified lesions assessed by IVOCT^5^. To predict the occurrence of stent under-expansion, Min et al. developed preprocedural intravascular ultrasound–based models^3^. Unlike intravascular ultrasound, IVOCT can penetrate the calcifications to visualize their thickness^4^, allowing for a more complete assessment of calcifications.

Our approach builds on our extensive experience in analyzing IVOCT images. Our group has applied machine learning^6, 7^ and deep learning^8–17^ methods on IVOCT images for segmentation and plaque characterization as well as for stent analysis^18–21^. Also, we have integrated computational fluid dynamics^22^ with finite element method^23^ to study the hemodynamic alternations following stenting.

In this report, we create an innovative machine learning method to predict stent deployment from pre-stent IVOCT images of calcified plaques. By using many lumen and calcification features and applying a segmental analysis to determine lumen area, we ensure that a regression model can accurately predict expansion. As the minimum stent expansion index (SEI) is used to classify under-expansions (SEI < 0.8), we compute SEIs and report classification predictions. As far as we know, this is the first time that such an automated, comprehensive machine learning approach has been applied to this important problem in interventional cardiology.‬‬ We compare our results to those from the current state-of-the-art.

## Methods

### Study population

This study was a retrospective single-center study conducted at the University Hospitals Cleveland Medical Center, Cleveland, Ohio, USA. IVOCT-guided PCI was performed in the cohort of all patients. From among 805 patients, we identified 104 patients. Exclusion criteria were (1) ostial lesion, (2) inability to cross the lesions with the OCT catheter because of tortuosity and/or occluding thrombus, (3) bypass graft stenosis, (4) in-stent restenosis, or (5) chronic total occlusion. In addition, lesions without either pre-stent or final OCT, without any calcium by OCT, or treated with plaque modification methods (i.e., rotational, laser, or orbital atherectomy or laser angioplasty) were excluded from this study. This retrospective study was approved by the Institutional Review Board (IRB) committee of University Hospitals Cleveland Medical Center (Cleveland, OH, USA). Written informed consent was waived by the Institutional Review Board of University Hospitals Cleveland Medical Center (Cleveland, OH, USA). All experiments were performed in accordance with relevant guidelines and regulations.

### Image acquisition and stent intervention

After the administration of 250 μg of intracoronary nitroglycerine, coronary angiography was performed with 6–7 F catheters through radial or femoral access. Angiograms were analyzed using QAngio® software (Medis, Leiden, the Netherlands). PCI was performed according to standard techniques. The choice of stent lengths and diameters was at the discretion of the interventionalist performing the procedure. Only drug-eluting stents were used in this study. OCT imaging was conducted using the C7XR FD-OCT Imaging System (Abbott Vascular, Santa Clara, CA, USA) after an injection of nitroglycerin (100–200 g). OCT was performed with a Dragonfly OPTIS 2.7 F 135-cm. Blood clearance was achieved by non-diluted iodine contrast using ISOVUE-370 (iopamidol injection, 370 mg iodine/mL; Bracco Diagnostics Inc., Princeton, NJ, USA). Images were acquired with an automated pullback at a rate of 36 mm/s using survey mode (375 frames, 75 mm), a frame rate of 180 frames/s, and an axial resolution of 20 μm. Images were deidentified and submitted to the Core Laboratory for independent offline analysis. Analysts who were blinded to the patient and procedural information in the Core Laboratory analyzed the OCT data. The reference lumen area was recorded by OCT automated measures or calculated by tracing the luminal contour on the proximal and distal reference segments.

### Machine learning model development

Our goal is to predict stent expansion, as assessed by minimal SEI, from pre-stent IVOCT images. Stents with minimum SEI (mSEI) ≤ or > 80% were classified as “under-expanded” and “well-expanded,” respectively. We achieved this by first predicting the poststent lumen area from the baseline images. The SEI for each frame is

$${\text{S}}\left( {\text{f}} \right) = \frac{{{\text{post}}\;{\text{stent}}\;{\text{lumen}}\;{\text{area }}\left( {\text{f}} \right)\;\left( {{\text{mm}}^{2} } \right)}}{{{\text{mean}}\;{\text{of }}\;{\text{proximal}}\;{\text{and}}\;{\text{distal}}\;{\text{references}}\left( {{\text{mm}}^{2} } \right)}}{*}100$$

The minimum SEI value (mSEI) is often reported as a metric of stent expansion. Consistent with the literature^1^, we deem a stent “under expanded” if the mSEI is ≤ 80% and “well expanded” if the mSEI is > 80%. Proximal and distal references were measured at the site with the largest lumen within 5 mm proximal and distal to the stented segment.

We adopted three approaches to predict under expansion: frame, segmental, and lesion. In the frame-based approach, we extracted features from each single-labeled frame to train a regression model to predict the poststent lumen area for each frame in a lesion. Features were from the two-dimensional lumen and calcification feature groups. In the segmental approach, we extracted features from a moving segment of image frames across the lesion. We applied moving segments with different lengths (i.e., 3, 7, 15, 31, and 63 frames) and a stride of 1 frame. The poststent lumen area was predicted for the central frame. In the lesion-based approach, all features were computed from the target lesion.

Patient data (110 lesions from 104 patients) were divided into a training data set (78 lesions) and held-out data set (32 lesions). Regression algorithms were developed using fivefold cross-validation across training data, where the training data were divided into internal training and test sets. Before testing on held-out data, we trained the regression models across all training data. Image processing and network training were performed using MATLAB software package (R2021a, MathWorks Inc.) on an NVIDIA GeForce TITAN RTX GPU with 120 GB of RAM installed in a Dell Precision T7610.

### IVOCT feature extraction and selection

Features were extracted using in-house developed software. Thirty-nine features, from four feature groups (12 two-dimensional lumen features, 6 three-dimensional lumen features, 12 two-dimensional calcification features, and 9 three-dimensional calcification features) were extracted. First-order aggregation statistics (minimum, maximum, mean, median, SD, skewness, and kurtosis) were obtained where applicable (e.g., for two-dimensional features in the case of the segmental approach). A total of 238 features were obtained (i.e., 168 two-dimensional features; 69 three-dimensional features). Feature values were normalized between 0 and 1. Other features such as lumen area were not normalized, because the absolute area is important. Table 1 summarizes the list of features. As appropriate, subsets of features were used for each of the frame, segmental, and lesion analysis approaches.

**Table 1:** List of the extracted features from each frame.

Lumen features		Calcification features		Statistics
2D features (Frame–based)	3D features (Lesion–based)	2D features (Frame–based)	3D features (Lesion–based)	
Area	Volume	Max arc angle	Volume	Mean
%Area of Stenosis	Equivalent diameter	Max thickness	Volume index	Median
Major axis length	Extent	Max depth	Length	SD
Minor axis length	Convex volume	Area	Equivalent diameter	Max
Perimeter	Solidity	Major axis length	Extent	Min
Extent	Surface area	Minor axis length	Convex volume	Skewness
Eccentricity		Extent	Solidity	Kurtoses
Solidity		Eccentricity	Surface area	
Circularity		Perimeter	Number of deposits	
Area < 0.5*Ref		Solidity	Calcification %	
Area < 0.7*Ref		Circularity		
Area < 0.9*Ref		Stretch ration		

This is a comprehensive list; recall that if you have an area, for example, we multiply it with the six statistical assessments. When we put all of this together, we get 238 features.

We used the least absolute shrinkage and selection operator (LASSO)^24^ for feature reduction in the regression models. This selection method applied a shrinking (regularization) process in which it assigned weights to regression variables. LASSO shrinks the regression coefficients toward 0 to eliminate irrelevant features from the regression model. In addition, we manually selected intuitively important features and statistics (highlighted in Table 1) to train the regression models. We called this intuitively selected group the calcification lesion expansion (CLE) group. We used LASSO to rank the CLE features based on their effects on the prediction. Calcification type (calcified nodule, calcified protrusion, and superficial calcific sheet) was used as an independent variable to examine the effect of calcification phenotypes on the performance of the lesion-based analysis.

These groups of features were then evaluated by using each of the machine learning regression models (decision tree, regression support vector machine, Gaussian process regression, and ensemble models). The poststent lumen area was predicted using these models, and then the associated SEI was computed. The root mean square error was the performance metric for the regression prediction. We used lumen area regression predictions to compute SEI(f) for a given lesion. We then searched the SEI(f) values to obtain the mSEI. Predictions were deemed under-expanded or well-expanded based on the definitions above. We compared our results with the method described by Fujino et al.^4^ for determining the stent deployment calcification score. The idea of calcification scoring is to define lesions that would benefit from plaque modification prior to stent implantation. The method is a cumulative score based on calcification: length, maximum angle, and maximum thickness. As quoting from their manuscript: “we assigned 1 or 2 points to each of three conditions: 2 points for maximum calcium angle > 180°, 1 point for maximum calcium thickness > 0.5 mm, and 1 point for calcium length > 5 mm.” In their study, they found that lesions with calcification score of 0 to 3 had “adequate stent expansion,” whereas lesions with a score of 4 had “poor stent expansion.” Classification performance was assessed using ROCs and confusion matrices with associated statistics.

### Statistical analysis

We used SPSS version 10.0 (SPSS, Chicago, IL, USA) to perform the statistical analyses to evaluate the patient and lesion characteristics at baseline (Supplementary Table S1). All values are expressed as mean ± SD (continuous variables) or as counts and percentages (categorical variables). Continuous variables were compared using unpaired Student *t*-tests, and categorical variables were compared using chi-square statistics. A *p* value of < 0.05 was considered to indicate statistical significance.

## Results

### Clinical data and processing

**Figure 1:**
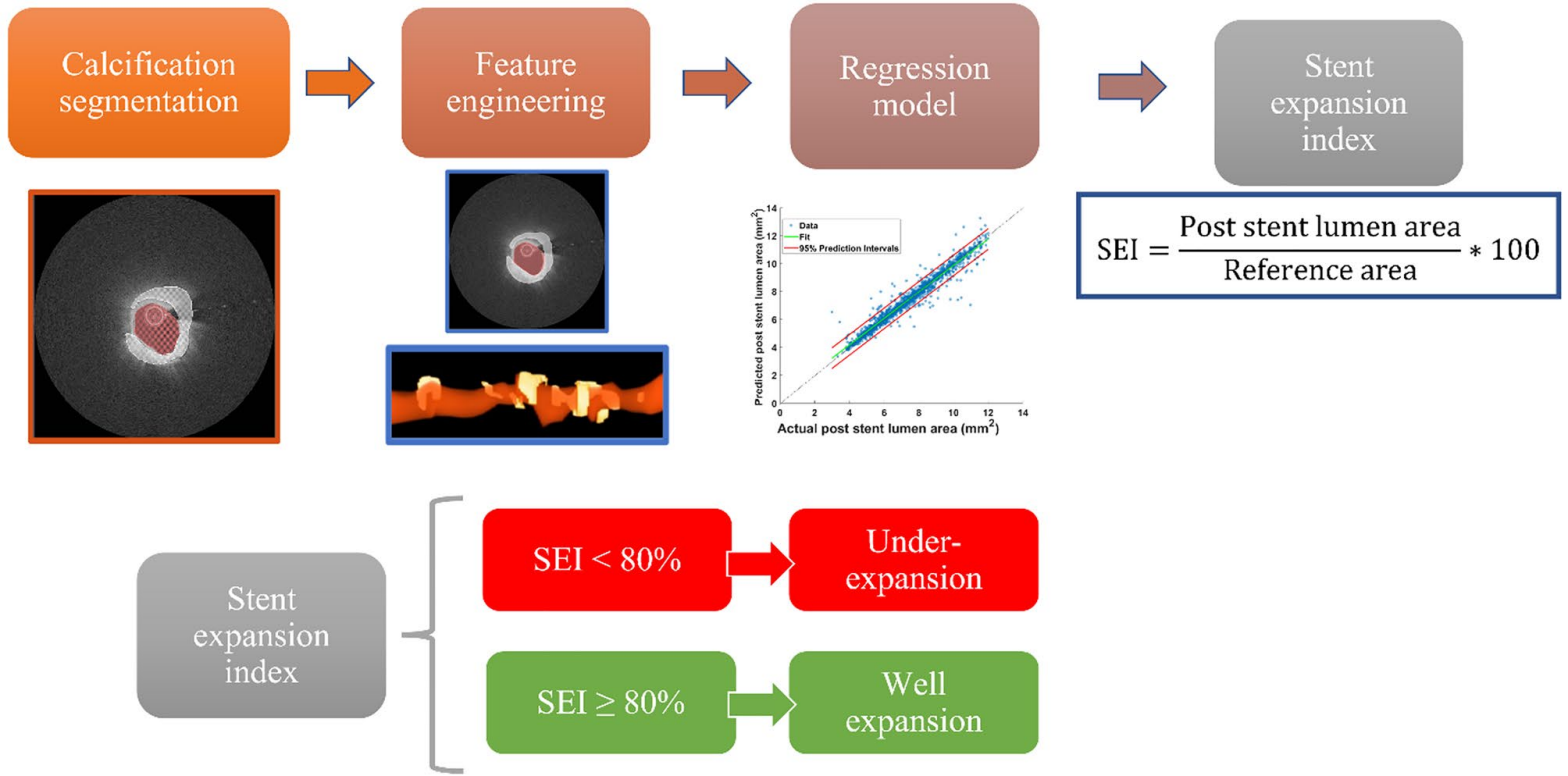
Prediction of stent under-expanded workflow. Features were extracted from the segmented image frames after automatic segmentation of both lumen and calcifications. Selected features were used to train regression models to predict the poststent lumen area along the vessel and compute the stent expansion index (SEI) for each stent. Stents with an SEI ≤ or > 80% were classified as “under-expanded” and “well-expanded,” respectively.

**Figure 2:**
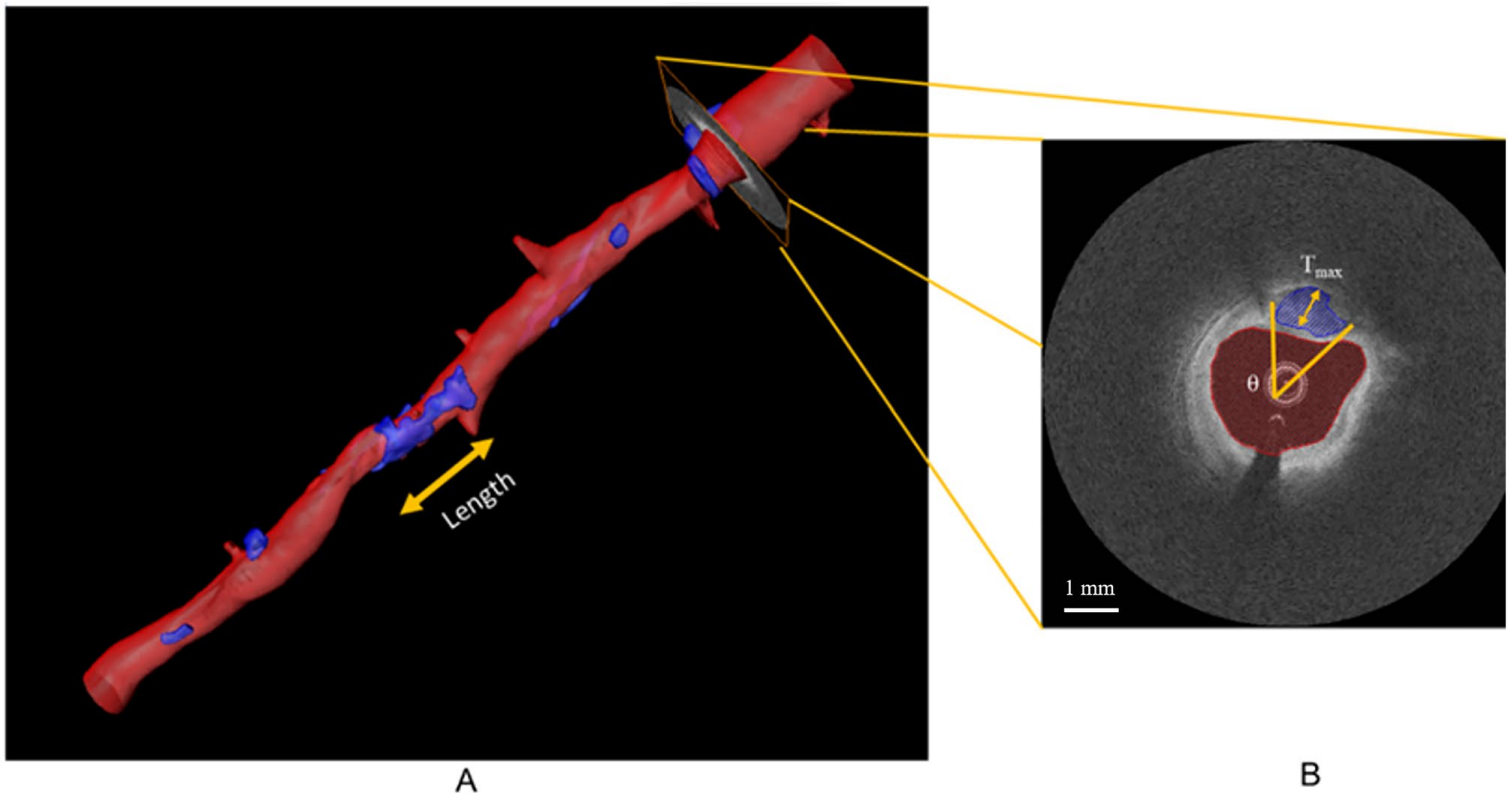
Calcifications and their quantification. The three-dimensional rendering (A) includes multiple calcifications in blue. In image slice (B), the calcification is tinted blue. Calcification attributes such as calcification length and calcification angle (θ) and maximum thickness (T_max_) can be measured from the three-dimensional volume and the two-dimensional image frame, respectively.

Supplementary Table S1 summarizes the baseline characteristics and procedural findings of the study cohort. There were 104 patients with 110 lesions: 55 were deemed under-expanded and 55 were deemed well-expanded, respectively. The overall flow of our approach is shown in Fig. 1. Supplementary Fig. S1 shows the registration of pre- and post-stenting IVOCT images. Supplementary Fig. S2 shows the results of the deep learning segmentation of calcifications. The extraction of calcification attributes is illustrated in Fig. 2.

### Performance of analysis methods

**Figure 3:**
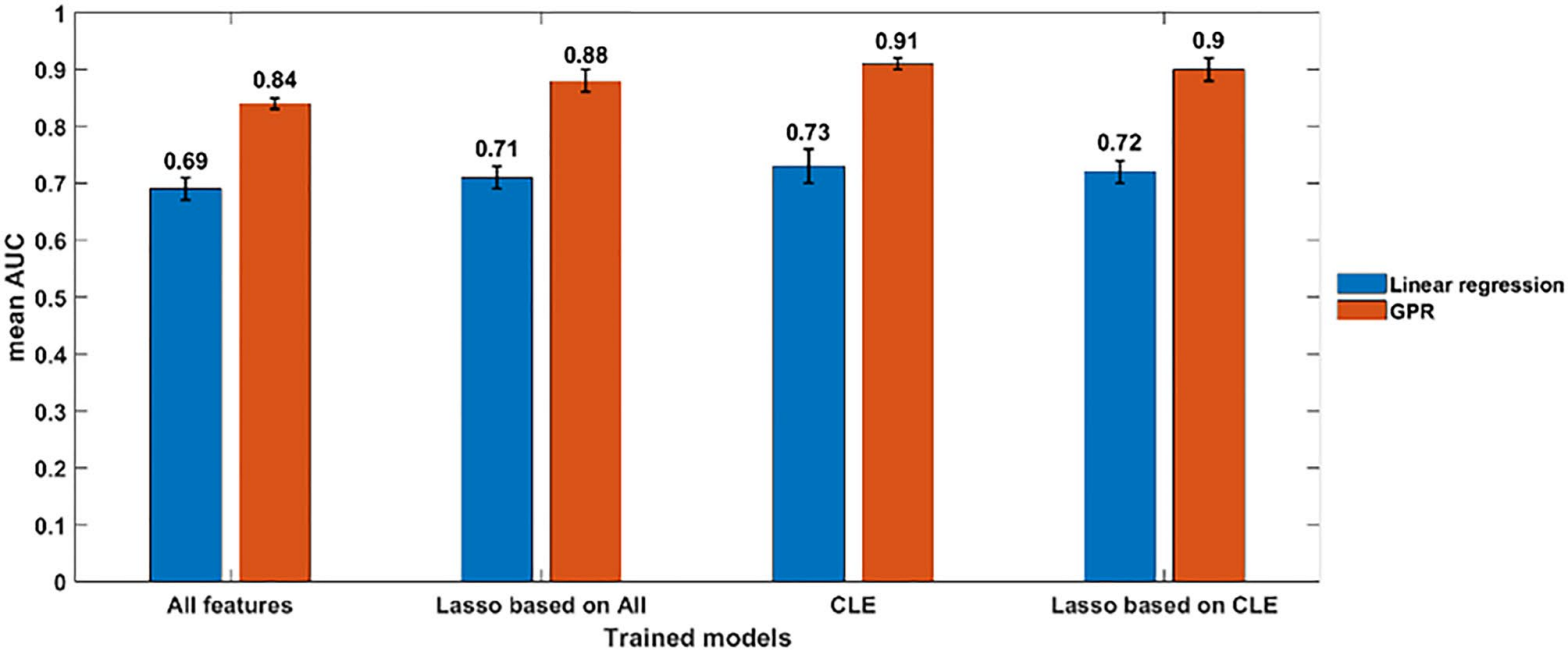
Comparison of linear regression (LR) and Gaussian process regression (GPR) as a function of different feature groups. The mean AUC was reported as the performance metric across the fivefold validation. Features selected based on LASSO improved the performance for both LR and GPR as compared with training both models using all features. A slight degradation in performance occurred when training both models using a subgroup of the best 20 features ranked by LASSO and based on the CLE feature group. The CLE features provided the best performance for both models.

We optimized the regression of predicted to actual areas after stenting. We used LASSO as a feature selection method. Figure 3 shows that the regression models for the segmental approach trained using the CLE set had the best performance. We compared two regression models, linear regression (LR) and Gaussian process regression (GPR), using four different feature sets (all features, LASSO features based on all features, CLE, and LASSO features based on CLE). The mean areas under the curve (AUCs) in fivefold cross-validation were reported. Features selected based on LASSO improved the performance for both LR and GPR compared with training both models using all features. We experienced a slight degradation in performance when training both models using a subgroup of the best 20 features ranked by LASSO and based on the CLE feature group. CLE features (features highlighted in Table 1) provided the best performance for both models. Table 2 shows the best 20 features of the CLE group ranked using LASSO.

**Table 2:** The CLE feature group ranked according to their importance as determined using LASSO on the segmental method.

#	Group	Feature	Statistic	#	Group	Feature	Statistic
1	Calcification	Angle	Mean	11	Calcification	Thick	Median
2	Lumen	Area	Mean	12	Calcification	Depth	Mean
3	Lumen	% AS	Median	13	Calcification	Thick	SD
4	Lumen	% AS	Mean	14	Lumen	Volume	–
5	Calcification	Area	Mean	15	Calcification	Depth	Median
6	Calcification	Angle	SD	16	Calcification	Thick	Mean
7	Lumen	% AS	SD	17	Calcification	Angle	Median
8	Calcification	%	–	18	Calcification	Area	Median
9	Calcification	Area	SD	19	Calcification	Volume	–
10	Lumen	Area	SD	20	Calcification	Depth	SD

The calcification angle, calcification area, lumen area, and percentage area stenosis (%AS) had the greatest impact on stent expansion. Calcification percentage (feature 8) is the percentage of frames with calcifications.

**Figure 4:**
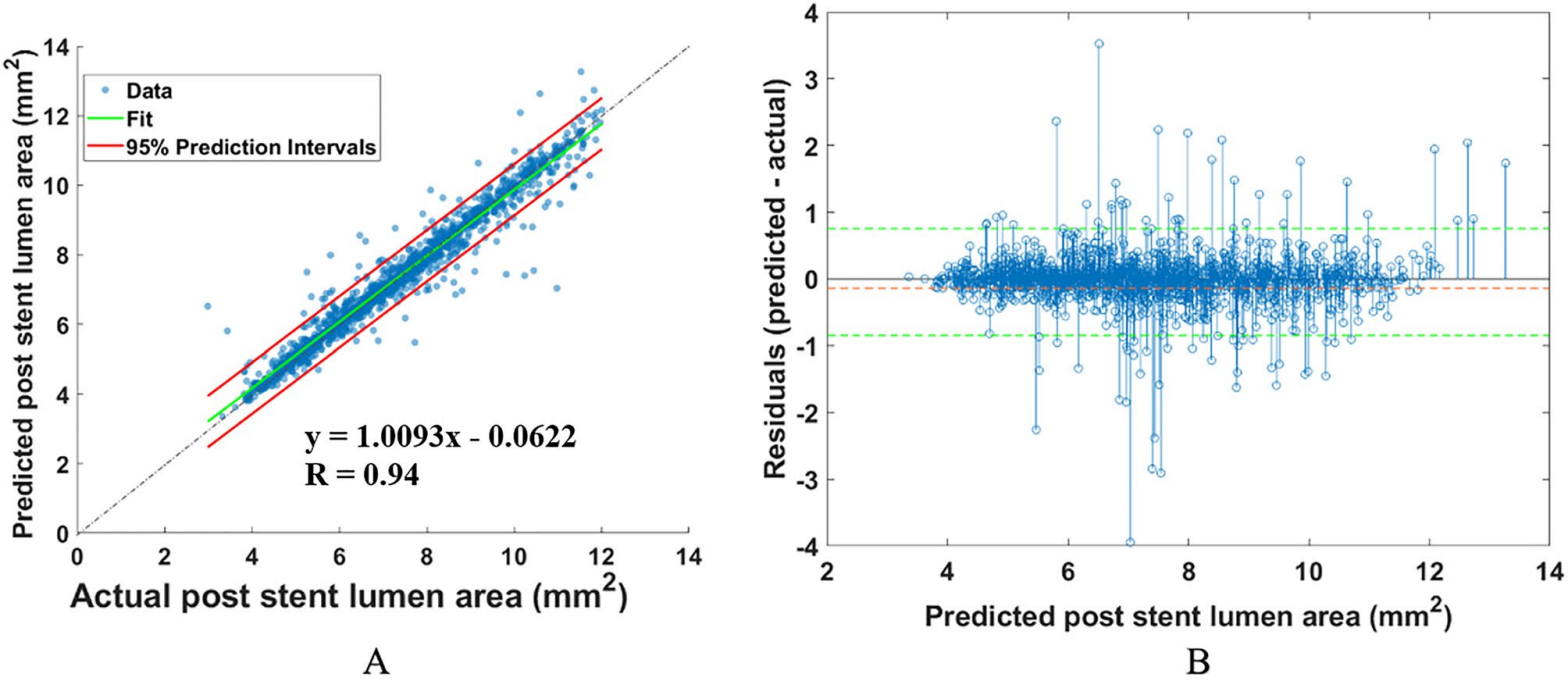
Regression prediction of the lumen area for the segmental analysis method. (**A**) The scatterplot shows a very high similarity (r = 0.94 ± 0.04) between the predicted and measured areas. (**B**) The residual plot yielded a small bias and reasonably small spread (− 0.1 ± 0.7 mm2).

Using our optimal method, the regression was quite good (Fig. 4). Figure 4A shows the excellent agreement against the actual measurements on the test data set (root-mean-square-error = 0.4 ± 0.02 mm^2^, *r* = 0.94 ± 0.04, *p* < 0.0001). Figure 4B shows the residual plot for the comparison between measured and predicted SEIs. Residual analysis indicated a very small bias (− 0.1 ± 0.7 mm^2^), and most of the measurements were included in the prediction interval.

**Figure 5:**
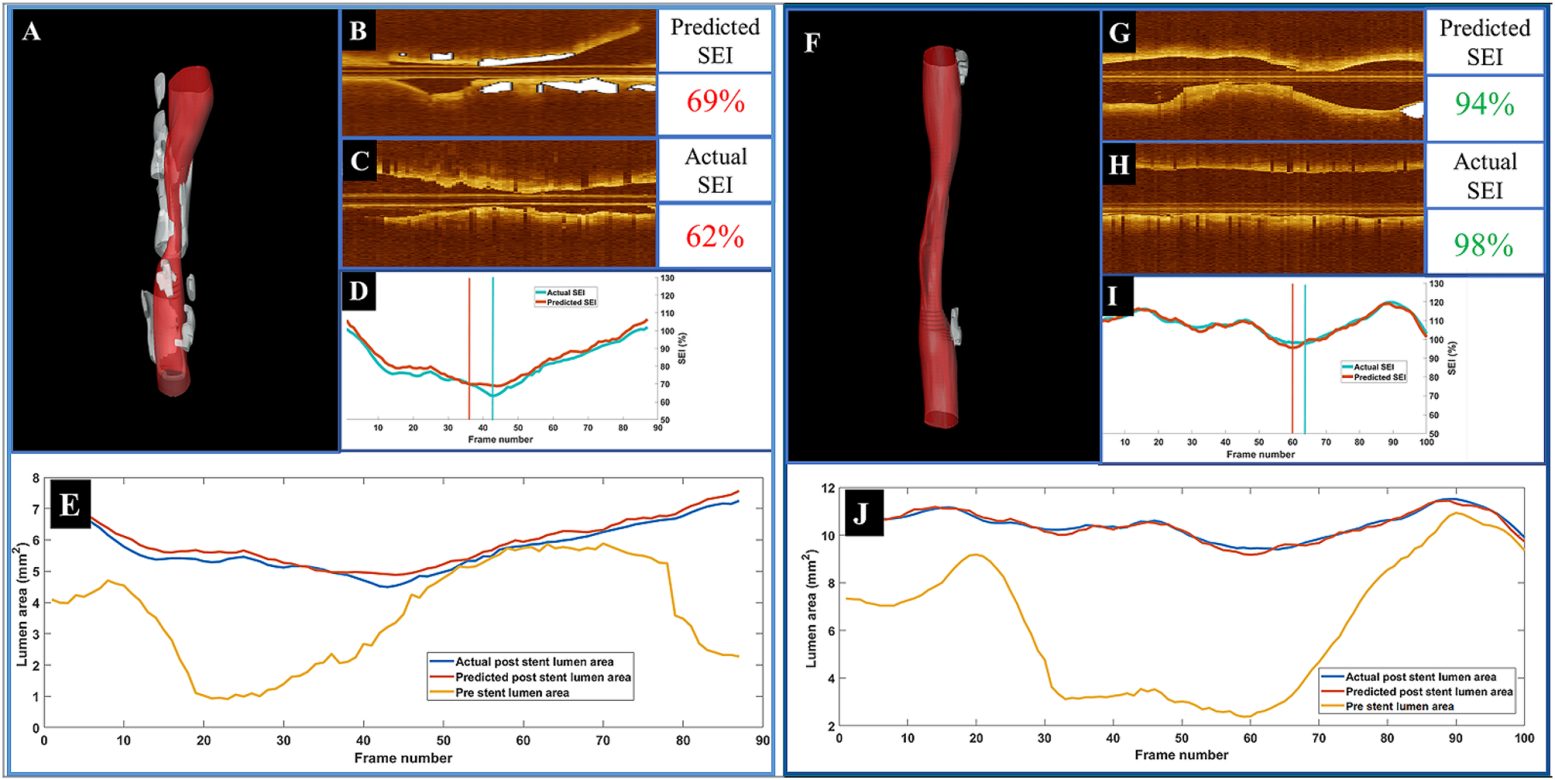
Predicted stent area in cases with different calcifications severity. Predicted stent area in a case of under-expansion in a heavily calcified lesion (left panel **A**–**E**) and a case with a well-expanded stent in a vessel with relatively little calcification (right panel **F**–**J**). (**A**, **F**) Three-dimensional rendering with calcifications in white, (**B**, **G**) longitudinal view before stenting with calcifications in white, (**C**, **H**) longitudinal view after stenting, and (**D**, **I**) predicted (orange) and actual (green) SEI following stenting. Our method predicted an SEI of 69%, which is close to the actual value of 62%, in which both values were indicative of under-expansion. The vertical bars in (**D**, **I**) show the locations corresponding to the minimum SEI values. The closeness of their location further suggests the predictive value of the regression model. The predicted and actual SEIs were 94 and 96%, respectively, and their locations were very close together. (**E** and **J**) show the effect of calcifications on stent expansion. Predicted and actual lumen areas after stenting are in blue and red, respectively. The orange curve represents the pre-stent lumen area for the registered pullback. In (**E**), areas were not enhanced after stenting because of the presence of calcifications (frames 50–70). (**J**) is associated with a well-expanded stent (frames 25–80).

It is enlightening to examine the actual and predicted lumen areas after stenting. Figure 5 shows two cases: an under-expansion case with a heavily calcified lesion (left panel) and one with a well-expanded stent in a vessel with mild calcification (right panel). The 3D visualization (Fig. 5A, F) and the longitudinal view (Fig. 5B, G) show calcification distribution. Figure 5C, H show the vessel after stent implantation. Predicted and actual lumen area curves (Fig. 5D, I) are remarkably close, and the predicted SEI is close to the actual. Applying the threshold of 0.8, both the prediction and actual measurement were classified as an under-expanded stent (left panel). The model predicted fairly closely the location of the frame with the minimum SEI. The right panel shows a well-expanded stent in the presence of little calcification. In this case, there was a remarkable agreement between the predicted and actual lumen area curves (Fig. 5I), and the SEIs were within 2%. Both the prediction and actual SEIs were consistent with a well-expanded stent. The effects of calcifications on stent expansion are depicted in Fig. 5E, J. Because of the presence of calcifications, the poststent lumen areas were not enhanced after stenting (frames 50–70) in Fig. 5E. Figure 5J is associated with a well-expanded stent (frames 25–80).

**Figure 6:**
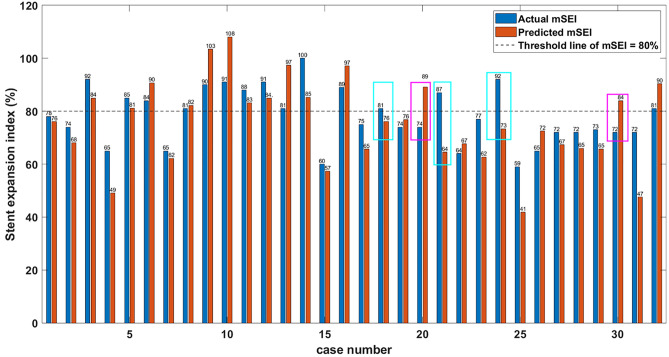
Predicted minimum SEI values (orange) are plotted with actual values (blue) for each stent in the test set. The horizontal line at 80% indicates the classification threshold for under-expanded. Our model successfully classified 27 of 32 cases in the test set. The cyan and magenta boxes indicate the five false-positive and false-negative cases for under-expanded, respectively. For two of the three false-positive cases, the prediction was sufficiently close to the threshold, such that a physician might override the prediction after reviewing the case. For the two false-negative cases in which the software did not predict the need for plaque modification, the actual minimum SEI was 0.72 or better, not far from the acceptable threshold of 0.8. In some cases, there was a discrepancy between minimum SEI values (e.g., case 25), but both were below the threshold of under-expanded. This difference is unimportant for clinical usage. After these considerations, there was only one gross error (case 21).

Figure 6 compares the actual minimum SEI (blue bars) and the predicted minimum SEI (orange bars) for each case in the test set. In this figure, the cyan and magenta boxes indicate the misclassification of five cases.

From the regression results, we obtained predicted SEI values and classified cases with stent under-expansion, corresponding to mSEI < 0.8. The values predicted by the frame-based approach showed good agreement against the actual measurements (root-mean-square-error = 0.12 ± 0.01 mm^2^, *r* = 0.63 ± 0.05, *p* < 0.0001, accuracy = 0.78 ± 0.04, and AUC = 0.79 ± 0.04). The performance of this approach exceeds the one by the lesion-based approach. For comparison, the lowest performance among all methods was that of the regression model at the lesion level (AUC = 0.73 ± 0.02). This is because of the small number of cases with this approach. Table 3 summarizes the performance of the different approaches.

**Table 3:** Summary of the performance of the analysis methods.

Metric	Frame-based	Segmental analysis	Lesion-based	Lesion-based with CP
Accuracy	0.78 ± 0.04	**0.84 ± 0.04**	0.71 ± 0.01	0.75 ± 0.02
Sensitivity	0.81 ± 0.06	**0.87 ± 0.05**	0.75 ± 0.01	0.75 ± 0.02
Specificity	0.75 ± 0.06	**0.82 ± 0.05**	0.68 ± 0.02	0.75 ± 0.01
AUC	0.79 ± 0.04	**0.85 ± 0.02**	*0.73* ± *0.02*	*0.76* ± *0.02*

The best performances were obtained by applying the segmental method (highlighted) with the Gaussian process regression (GPR) algorithm in combination with the CLE feature group. We also examined the effect of adding the calcification phenotype as an independent variable on the performance of the lesion-based model. We retrained the models of the lesion-based approach after adding the calcification type as an independent variable. The mean AUC was improved from 0.73 to 0.76, as indicated in the italics.

Significant values are in bold.

### Comparison with the existing state-of-the-art techniques

**Figure 7:**
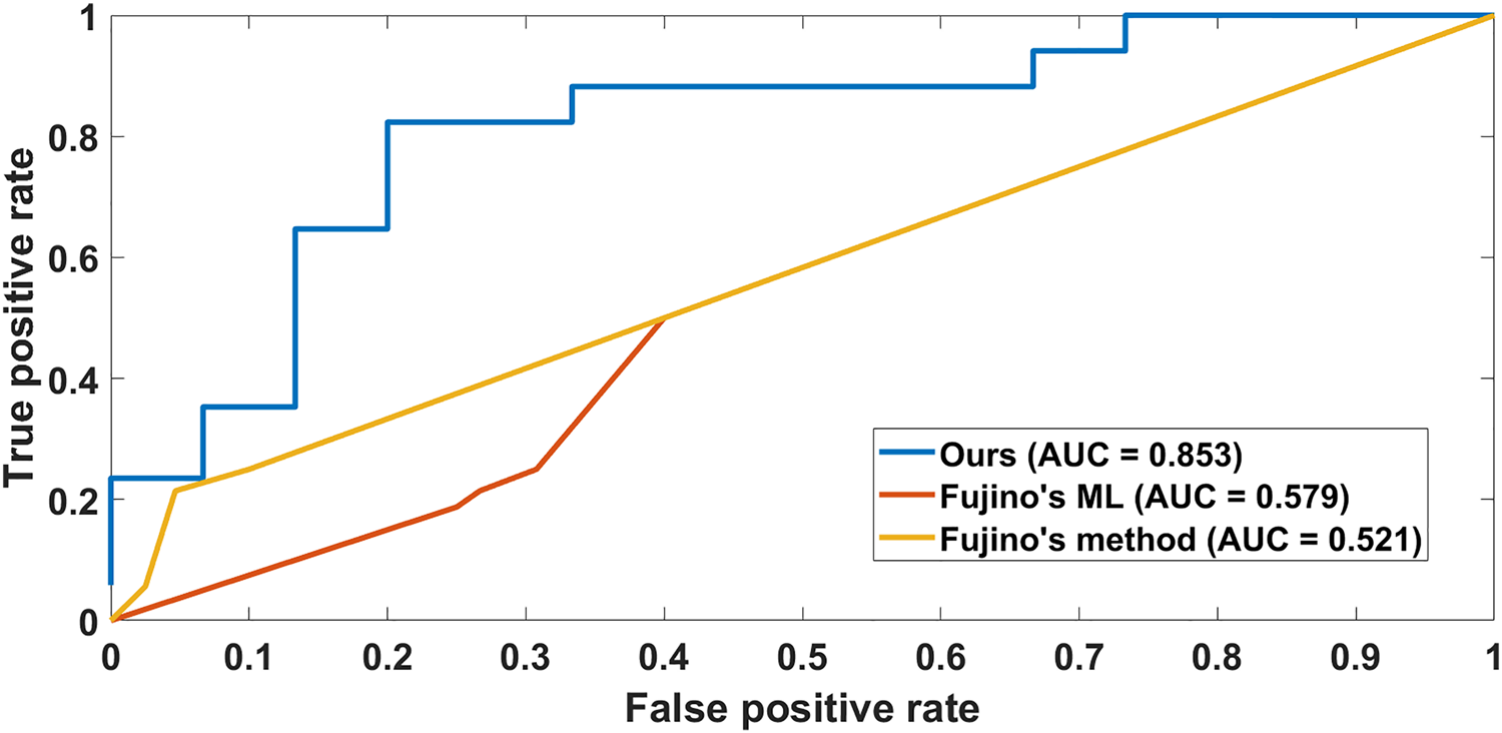
Receiver-operating characteristic curves comparing the performance of our method to the state-of-the-art method reported by Fujino et al.^4^. Our method, Fujino’s method as reported in 4, and Fujino’s ML are shown. The AUCs were 0.853 (our method), 0.58 (Fujino’s ML), and 0.52 (Fujino’s method). Our method provided significantly improved prediction as compared with their method (*p* < 0.005).

Our optimized segmental method provided significantly better prediction as compared with the seminal work by Fujino^4^. Figure 7 shows the receiver-operating characteristic curves for our method, Fujino’s method, and Fujino’s machine learning (ML) where we used features from their publication and applied machine learning to our data. Across the folds, our method achieved a mean accuracy of 0.84 ± 0.03 and a mean AUC of 0.85 ± 0.02 after converting the regression problem to the classification problem using an SEI of 0.8 as a cutoff value. In the figure, the mean AUCs were 0.85 (our method), 0.58 (Fujino’s ML), and 0.52 (Fujino’s method). Our method provided superior results with a significant difference (*p* < 0.005) as compared to the other methods.

### Effect of calcification phenotype on lesion-based prediction

**Figure 8:**
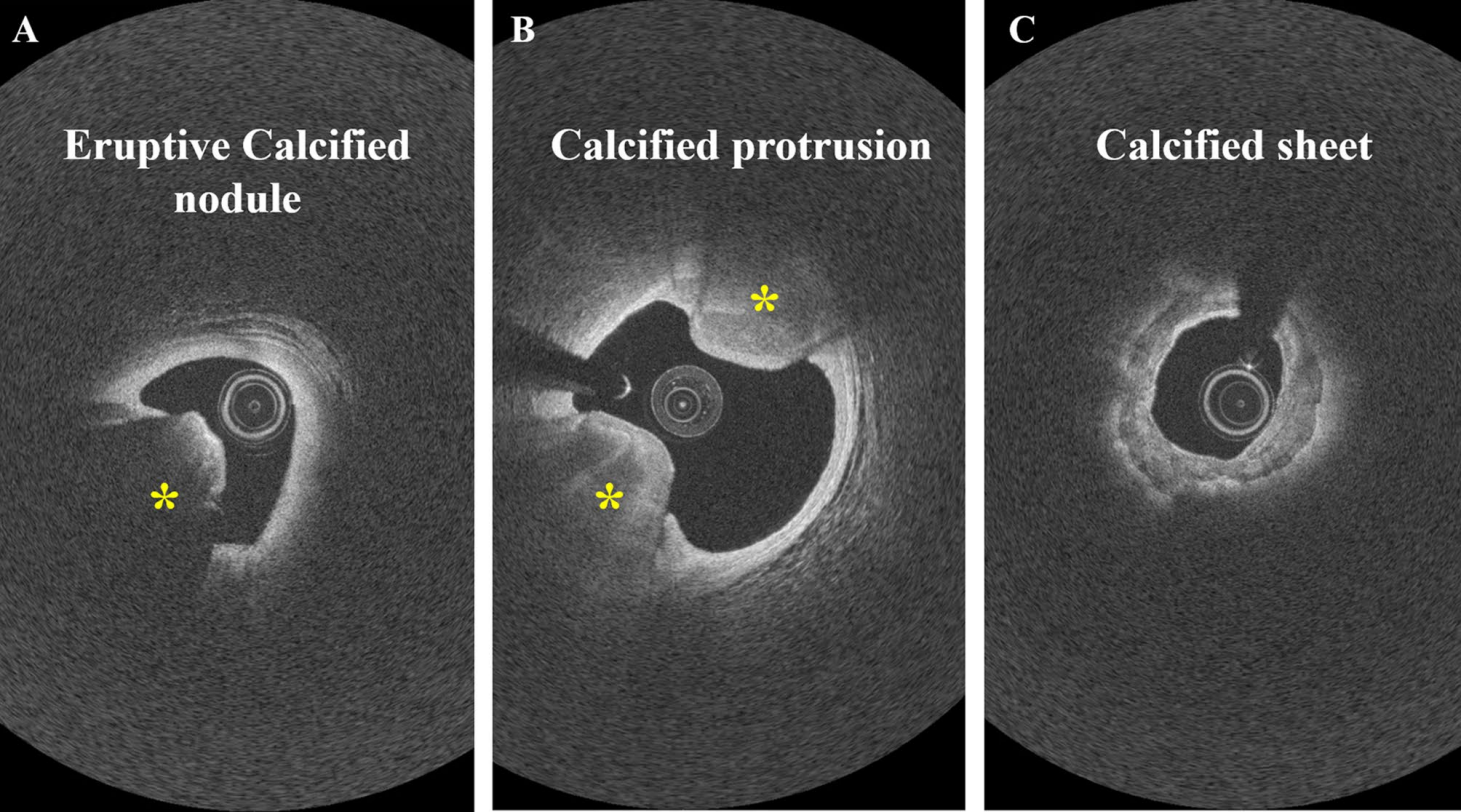
Calcification types in the IVOCT images. (**A**) Eruptive calcified nodule. (**B**) Calcified protrusion. (C) Superficial calcific sheet.

We investigated the impact of calcification phenotype on stent expansion using our lesion-specific analysis. Our data included three types of calcifications based on the reports in the literature^25, 26^: eruptive calcified nodule (~ 13%), calcified protrusion (~ 23%), and superficial calcific sheet (~ 64%). In IVOCT images, the eruptive calcified nodule has an erupted volcanic shape with clusters of small calcific nodules that protrude into the lumen (Fig. 8A). Eruptive calcified nodules are dominant in heavily calcified coronary arteries. Eruption of calcific nodules causes disruption of the endothelium that is associated with red thrombus. Calcified protrusion also protrudes into the lumen but without eruptive nodules (Fig. 8B). IVOCT-derived eccentric shape of the protruding mass could induce local flow disturbance and high endothelial shear stress, resulting in thrombus formation. The leading edge of eruptive calcified nodules is irregular due to a cluster of small calcific nodules, whereas calcified protrusions show a smooth leading edge. The calcific sheet is superficial (i.e., very close to the lumen border) with a minimum overlying fibrous layer. It has minimal or no protrusion in the lumen, maintaining the circle-like shape of the lumen (Fig. 8C). Luminal narrowing due to the calcific sheet creates a local high endothelial shear stress environment that lead to thrombosis.

**Figure 9:**
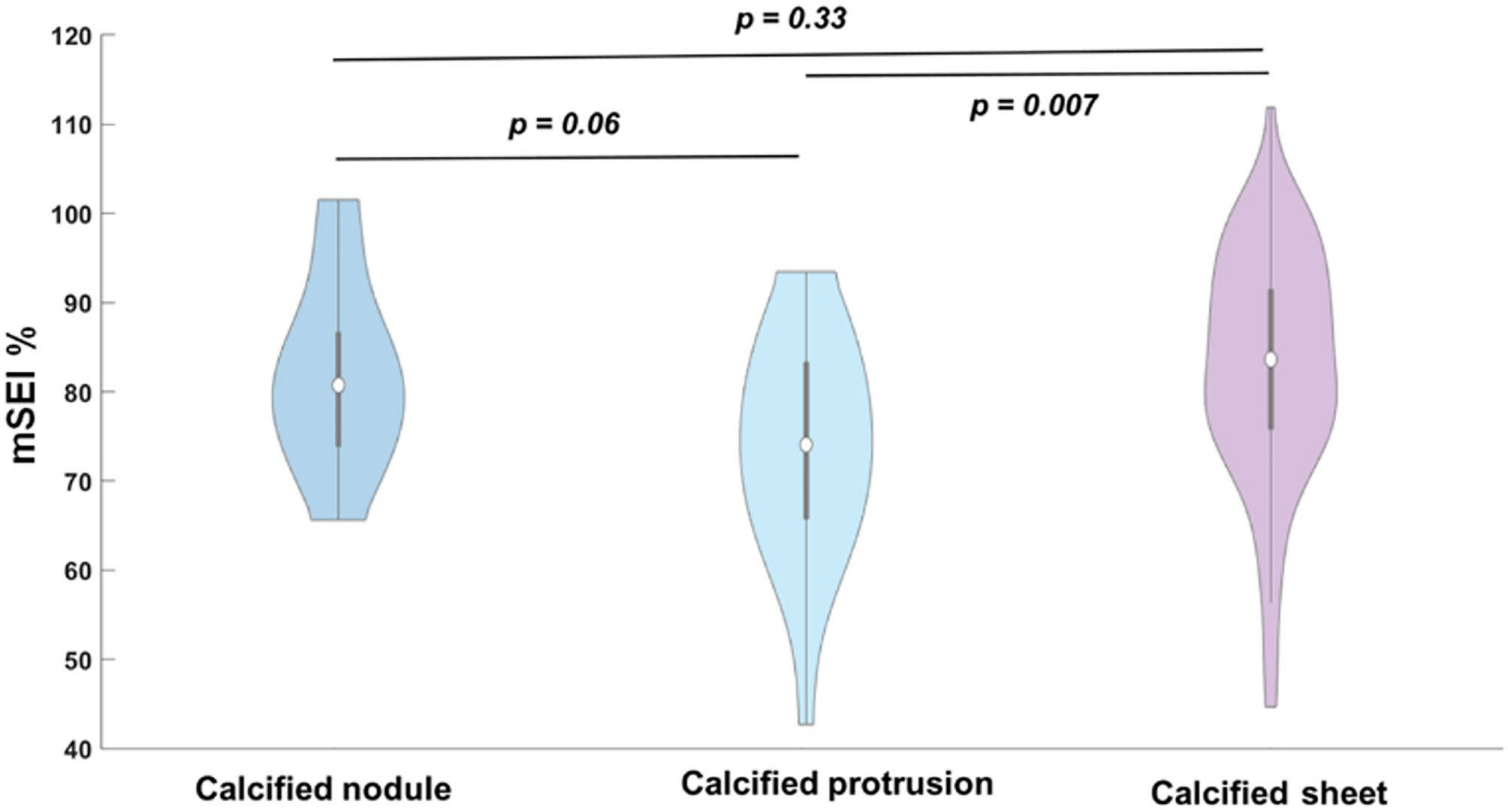
Distribution of the SEI among different calcification phenotypes. Calcification protrusion had the lowest mSEI median value as compared with the other types.

The calcification phenotype was assigned by visual examination of the frame with the mSEI in the lesion. Figure 9 shows the mSEI values for the three phenotypes of the calcified lesions. To predict a well-expanded stent, we retrained the lesion-specific model by adding calcification phenotype as an independent variable. As indicated by the red box in Table 3, the AUC improved from 0.73 ± 0.02 to 0.76 ± 0.02.

## Discussion

Accurate prediction of stent deployment with suboptimal expansion using pre-procedural imaging may allow for the identification of patients who are more likely to have adverse outcomes. In this work, we developed machine learning methods to predict stent under expansion of coronary artery using baseline IVOCT images.

To our knowledge, this is the first IVOCT-based machine learning assessment of stent underexpansion. The main findings of the present study were as follows: (1) segmental approach has the best performance with mean AUC of 0.91; (2) using manually selected features, CLE feature group, with both lumen and calcification attributes enhanced the prediction of stent under-expansion; (3) plaque characteristics were automatically computed using deep learning; and (4) adding calcification type as a risk factor enhanced the prediction performance at the lesion-level. We also investigate the effect of using different feature selection methods.

Our segmental method very well predicts vessel expansion after stenting from IVOCT images taken before stenting. Lumen areas are predicted remarkably well along the full length of a vessel (Fig. 5), giving an accurate SEI prediction for these cases. When we analyzed misclassifications in detail (Fig. 6), we found only one gross error (case 21) out of 32 cases where the predicted minimum SEI (0.64) predicted under-expansion when the actual result was 0.87, indicating a true adequate expansion. Of course, in any implementation, a physician would make the final determination of how to treat a lesion. SEIs are predicted well (Fig. 5), and stent under expansion (SEI < 0.8) is predicted with an accuracy of 0.84 ± 0.04 (Table 3) and AUC of 0.85 (Fig. 7).

The ability to accurately predict results after stenting from images taken before stenting is significant. Once a stent is deployed in the atherosclerotic tissue, it is very difficult or impossible to increase the expansion, even with a post-dilation balloon under high pressure. Hence, the accurate prediction of stent deployment from pre-stent imaging is very important when planning intervention treatment. Some of the options for lesion preparation include rotational and orbital atherectomy, cutting or scoring the balloon, acoustic shock wave, and/or balloon pre-dilation. Because special devices are costly and carry some potential risks, the precise prediction of their need is advisable.

The segmental approach provided much better performance than single-frame or lesion approaches did. This might be because the segmental approach inherently includes many more instances of regression learning as compared with the lesion-based approach. With many more training samples, the difference in performance might be reduced. The segmental approach performed better than the single-frame approach did, likely because a single-frame does not capture the effect of the extent of calcification. This rationale is evident in the optimal frame length consisting of 31 frames or 6.2 mm. We infer that smaller segmental lengths did not account for the full local biomechanics, and longer segments might have required more training samples. Although we tried multiple feature reduction techniques, we found that our manually selected features, the CLE feature group, consisting of both lumen and calcification attributes, provided the best predictions. Adding the calcification phenotype as a risk factor enhanced the prediction performance of the lesion-based method. When we applied different feature selection techniques, calcification phenotype never emerged as a top-ranked feature for segmental- and frame-based models, potentially because other features captured these characteristics.

It is instructive to examine the most important features for predicting stent deployment. Table 2 shows the top 20 CLE features ranked by LASSO for the prediction of stent under-expansion. The most important predictors are the calcification angle and area, as well as the lumen area and percentage area stenosis. Figure 3 shows the impact of using features selected by LASSO to train regression models using the segmental analysis approach. First, we trained the LR model and GPR using the whole feature set. Then we applied LASSO to rank the best features and used them to train the same models. LASSO successfully improved the performance of both models. Training both models using the CLE features provided the best performance. After using features from the CLE group and selected by LASSO, the performance for both models was slightly degraded. The prediction of stent expansion is a complicated process that requires the inclusion of many parameters during the training phase.

We can compare our study to that of the groundbreaking study of Fujino et al.^4^. Those authors created an IVOCT-based calcium scoring system that can be used to predict stent under-expansion in which scoring was based on a simplified analysis of the maximum angle, maximum thickness, and length of calcification. Our more complex approach provided significantly better prediction as compared with both their method and their features in a machine learning approach (Fig. 7). They determined calcification attributes in a semiautomated manner, whereas our method is fully automated based on deep learning segmentation, providing results within seconds.

The study has limitations. It will be important to reproduce these results on larger data sets. Further validation across multi-institutions would be needed to establish the robustness of this machine learning-based prediction approach. Only shape features were extracted from the lumen and calcification region. Extending the features set to include another type of features (i.e., radiomics features) and/or another type of lesion (i.e., fiber tissue) may improve the prediction performance.

In conclusion, machine learning approaches can predict stent deployment. Because it is difficult or impossible to correct stent under-expansion after the stent is deployed, a method to predict stent deployment from pre-stent images is important when planning the intervention.

### Supplementary Information

Supplementary Information.

## Data Availability

The datasets analyzed during the current study are available from the corresponding author upon reasonable request.
