# Supplementary material for: Prediction of stent under-expansion in calcified coronary arteries using machine learning on intravascular optical coherence tomography images
Source: Sci Rep. 2023 Oct 23;13:18110. doi: 10.1038/s41598-023-44610-9 (PMC10593923; doi:10.1038/s41598-023-44610-9)
Supplement: Supplementary file 1 — Supplementary Information. [file 41598_2023_44610_MOESM1_ESM.pdf]

SUPPLEMENTARY MATERIALS

Supplemental Table S1. Baseline Patient Characteristics.

	**Overall n = 104 (100%)**	**Training /testing set n=72/104 (69.2%)**	**Validation set n=32/104 (30.8%)**	**p-value**
**Patient-level characteristics**	**Mean ± SD (N) (Min, Max)**			
Age (years)	67.1±12.0 (104) [30, 98]	67.6±12.4 (72) [40,98]	65.9±10.9 (32 [30, 85])	0.494
**Gender**				
Male	74/104 (71.15%)	47/72 (65.3%)	27/32 (84.4%)	
**Physical Measurement**				
Height (cm)	171.8 ± 9.8 (104) [144.8, 195]	171.3±9.6 (72) [152.4, 195]	172.8±10.1 (32) [144.8, 190.5]	0.482
Weight (kg)	93.7 ± 25.3 (104) [40.8, 225]	91.1±18.5 (72) [51.7, 151]	99.0±35.9 (32) [40.8, 225]	0.257
BMI (kg/m^2)	31.73 ± 8.1 (104) [17.9, 75.2]	31.2±6.3 (72) [17.9, 49.1]	32.9±11.3 (32) [19.4, 75.2]	0.426
**Medical History**				
Hypertension	99/104 (95.19%)	69/72 (95.8%)	30/32 (93.7%)	0.676
Diabetes Mellitus	56/104 (53.84%)	39/72 (54.2%)	17/32 (53.1%)	0.923
Hyperlipidemia	90/104 (86.54%)	63/72 (87.5%)	27/32 (84.4%)	0.683
Previous PCI	8/104 (7.69%)	6/72 (8.3%)	2/32 (6.2%)	
Previous Myocardial Infarction	60/104 (57.69%)	42/72 (58.3%)	18/32 (56.5%)	0.062
Heart Failure, LVEF <30%	58/104 (55.77%)	41/72 (56.9%)	17/32 (53.1%)	0.723
Previous CABG	8/104 (7.69%)	6/72 (8.3%)	2/32 (6.2%)	0.703
Current Smoker (≤6 Months)	53/104 (50.96%)	40/72 (55.5%)	13/32 (40.6%)	0.165
Renal Dysfunction (Serum Creatinine > 2.0)	53/104 (50.96%)	34/72 (47.2%)	19/32 (59.4%)	
Hemodialysis or Renal Transplant	12/104 (11.54%)	9/72 (12.5%)	3/32 (9.4%)	
**Pre-procedure Presentation**				
STEMI/Cardiogenic shock	10/104 (9.6%)	6/72 (8.3%)	4/32 (12.5%)	
NSTEMI/Unstable Angina	35/104 (33.65%)	24/72 (33.3%)	11/32 (34.4%)	
Stable Angina	57/104 (54.81%)	41/72 (56.9%)	16/32 (50.0%)	
Silent Ischemia	0/104 (0.0%)	0/72 (0.0%)	0/32 (0.0%)	
Other*	1/104 (0.96%)	1/72 (1.4%)	0/32 (0.0%)	

*Aortic stenosis.

Registration and Segmentation

The cross-sectional IVOCT frames between pre- and poststent pullbacks were co-registered frame by frame by using the landmarks such as the ostium, the opening of the side branch, coronary vein, calcifications, and other representative structures, as shown in Supplementary Figure 1. In Supplementary Figure 2, both the lumen and calcifications were segmented using our previous deep learning-based segmentation method [12]. Lumen and calcification labels were then used as the inputs of the feature extraction process.

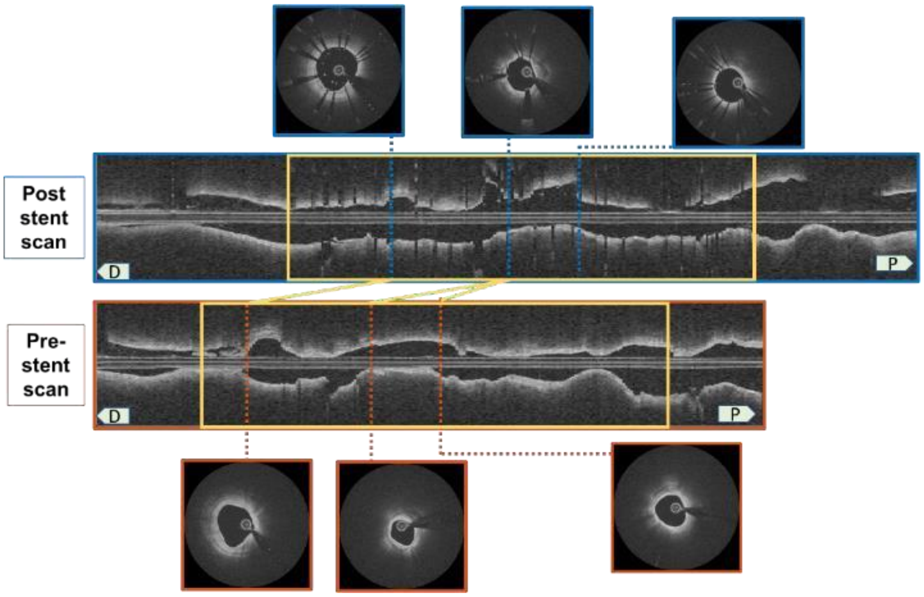

Supplementary Figure S1. Co-registration of pre- and post-stenting IVOCT images. This is a manual process whereby landmarks (e.g., side branches and calcifications) were matched up by changing the z-offset and angle of the poststent image.

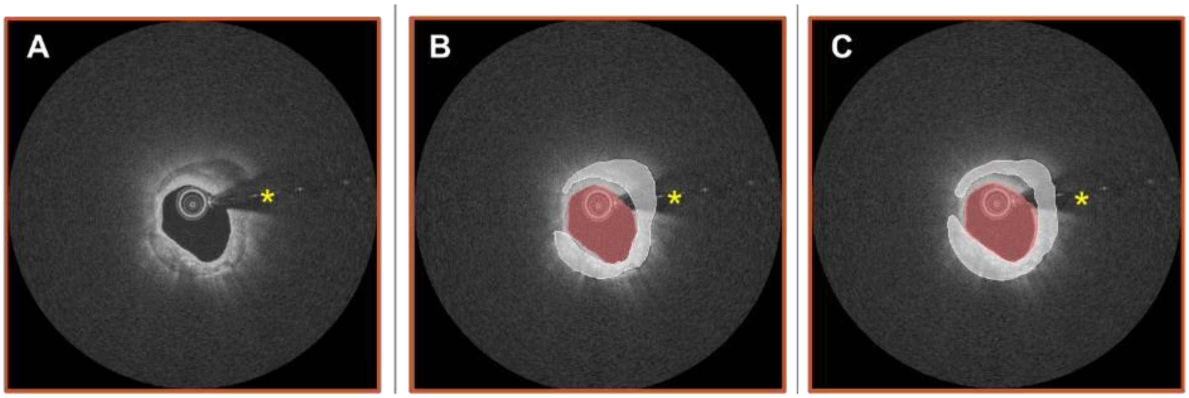

Supplementary Figure S2. IVOCT automated segmentation using our deep learning approach, described in (12). (A) IVOCT image. (B) Manually labeled image. (C) Automatic segmentation. The lumen is shown in red, and the calcification is shown in white. There was a very good agreement between manual and automated assessments. The asterisk (*) indicates the guidewire shadow.
